# ARPC5 acts as a potential prognostic biomarker that is associated with cell proliferation, migration and immune infiltrate in gliomas

**DOI:** 10.1186/s12885-023-11433-w

**Published:** 2023-10-03

**Authors:** Yue Ming, Chunyuan Luo, Beihong Ji, Jian Cheng

**Affiliations:** 1 Laboratory of Molecular Oncology, Frontiers Science Center for Disease-related Molecular Networks, West China Hospital, Sichuan University, Chengdu, Sichuan China; 2 Department of Pharmaceutical Sciences, School of Pharmacy, University of Pittsburgh, Pennsylvania, USA; 3 Department of Neurosurgery, West China Hospital, Sichuan University, Chengdu, Sichuan China

**Keywords:** ARPC5, Glioma, Prognosis, Tumor immunity, Biomarker

## Abstract

**Background:**

Gliomas are the most common malignant brain tumors, with powerful invasiveness and an undesirable prognosis. Actin related protein 2/3 complex subunit 5 (ARPC5) encodes a component of the Arp2/3 protein complex, which plays a significant role in regulating the actin cytoskeleton. However, the prognostic values and biological functions of ARPC5 in gliomas remain unclear.

**Methods:**

Based on the TCGA, GEO, HPA, and UALCAN database, we determined the expression of ARPC5 in glioma. The results were verified by immunohistochemistry and Western blot analysis of glioma samples. Moreover, Kaplan-Meier curves, ROC curves, Cox regression analyses, and prognostic nomograms were used to observe the correlation between the ARPC5 expression and the prognosis of glioma patients. GO and KEGG enrichment analyses were conducted to identify immune-related pathways involved with the differential expression of ARPC5. Subsequently, the TCGA database was used to estimate the relationship between ARPC5 expression and immunity-related indexes, such as immune scores, infiltrating immune cells, and TMB. The TCIA database was used to assess the correlation between ARPC5 with immunotherapy. The association between ARPC5 and T cells marker CD3 was also evaluated through immunohistochemistry methods. The correlation between ARPC5 and T cell, as well as the prognosis of patients, was also evaluated using immunological methods. Moreover, the effect of ARPC5 on the biological characteristics of LN229 and U251 cells was determined by MTT, clone formation, and transwell migration assay.

**Results:**

The high degree of ARPC5 was correlated with worse prognosis and unfavorable clinical characteristics of glioma patients. In the analysis of GO and KEGG, it is shown that ARPC5 was strongly correlated with multiple immune-related signaling pathways. The single-cell analysis revealed that ARPC5 expression was increased in astrocytes, monocytes and T cells. In addition, ARPC5 expression was strongly associated with immune scores, infiltrating immune cells, TMB, MSI, immune biomarkers, and immunotherapy. In experimental analysis, we found that ARPC5 was significantly overexpressed in gliomas and closely correlated with patient prognosis and CD3 expression. Functionally, the knockout of ARPC5 significantly reduced the proliferation and invasion of LN229 and U251 cells.

**Conclusions:**

Our study revealed that the high expression level of ARPC5 may serve as a promising prognostic biomarker and be associated with tumor immunity in glioma.

**Supplementary Information:**

The online version contains supplementary material available at 10.1186/s12885-023-11433-w.

## Introduction

Gliomas are the most widespread intracranial primary tumors that result in severe mortality and poor prognosis, accounting for approximately 80% of malignant brain tumors [1, 2]. With the increasing number of molecular targets applied in clinical diagnosis and therapy, the classification of gliomas has changed. The 2016 World Health Organization (WHO) classification of central nervous system (CNS) tumors combined with histologic features and molecular characteristics for the first time to promote refinement of tumor classification, such as IDH mutation, 1p/19q codeletion, and MGMTp methylation [3, 4]. According to the 2021 revised fifth edition of the WHO classification, several significant changes have been made in the principles relating to nomenclature, grading, and classification of CNS tumors [5]. Moreover, these shifts will increase the dependence on molecular changes in disease classification and increase the importance of molecular detection in clinical applications. Thus, we develop new glioma biomarkers to offer more diagnostic and therapeutic opportunities in clinical practice.

Actin-related protein 2/3 (Arp2/3) complex is a well-known actin nucleator that can promote the actin branched junction, which may be a crucial participant in the migration and invasion of various cancers [6]. Previous studies showed that the Arp2/3 complex may be a key player in glioma cell invasion and migration, and may represent a new target for the treatment of glioma [7]. ARPC5, a member of the Arp2/3 complex, was reported to influence many critical biological processes in cancer, such as cell migration, invasion, and differentiation [8, 9]. For example, ARPC5 played as a pro-metastasis gene in prostate cancer and exhibited a negative regulation with miR-141 [10]. Moreover, some researchers found that ARPC5 was an independent predictor of hepatocellular carcinoma, and its expression had significantly positively correlation with the infiltration of immune cells [11]. However, the association between ARPC5 expression and clinical significance in glioma is still unclear.

In our study, we explored the expression and clinical significance of ARPC5 in gliomas by integrating multiple databases and in-depth assessing its impact glioma.

## Materials and methods

### Data collection

The Tumor Immune Estimation Resource (TIMER, http://timer.cistrome.org/) was used to analyze the expression levels of ARPC5 between tumor tissues and adjacent normal tissues across all The Cancer Genome Atlas (TCGA, https://portal.gdc.cancer.gov/) tumors [12]. The Gene Expression Profiling Interactive Analysis (GEPIA, http://gepia2.cancer-pku.cn/#analysis) is an interactive online analysis platform that contains abundant human specimens from TCGA and GTEx database [13]. Based on this platform, we analyzed the differential expression of ARPC5 between glioma tissues and matched normal brain tissues. The Gene Expression Omnibus (GEO) contains high-throughput gene expression data in various cancer. To explore ARPC5 expression levels in glioma, we examined microarray data from three datasets: GSE2223 (glioma = 50, normal = 4), GSE29796 (glioma = 52, normal = 20), and GSE116520 (glioma = 34, normal = 8). The RNA sequences and their corresponding clinical data from 1,018 samples were obtained from the Chinese Glioma Genome Atlas (CGGA, http://www.cgga.org.cn/index.jsp). Additionally, we analyzed TCGA to obtain the expression sequences and clinical information of gliomas, including 5 normal samples and 701 tumor samples. The Human Protein Atlas (HPA, https://www.proteinatlas.org/), and the University of Alabama at Birmingham Cancer data analysis Portal (UALCAN, https://ualcan.path.uab.edu/) were used to analyze the protein expression levels of ARPC5 in gliomas [14]. The processed single-cell data were downloaded from the CGGA database [15]. All data Accessed on 27 March 2023. Before further analysis, all sample databases were screened to exclude samples with unknown or incomplete clinicopathological data.

### Analysis of clinical characteristic and prognosis of ARPC5 in glioma

The correlation between ARPC5 expression and clinical characteristic from CGGA database was analyzed using R packages “beeswarm”. Kaplan-Meier survival analysis was used to assess the overall survival for glioma. Patients were classified into high- and low-expression groups by the median value of ARPC5 expression, and then analyzed the subgroups of glioma cases, such as different grade, gender, age, IDH mutation status, 1p/19q codeletion status, and MGMTp methylation status. Survival curves were drawn using the R packages “survival” and “survminer”. Then, we calculated the ROC curve of ARPC5 at 1-, 3-, and 5-years through R packages “survival ROC”. Moreover, the clinical prognostic value of ARPC5 was assessed by univariate along and multivariate Cox regression models. Finally, we established a nomogram model containing ARPC5 expression and various clinical characteristics to predict the survival prognosis of glioma patients in 1, 3, and 5 years, based on the CGGA database. The nomogram was drawn using the R packages “survival” and “rms”.

### Gene set enrichment analysis (GSEA)

GSEA analysis was conducted based on the gene sets of “c5.go.v7.4.symbols” and “c2.cp.kegg.v7.4.symbols” in CGGA database. Divide the samples in the CGGA database into high and low expression groups, based on the median scores of ARPC5 expression. Under the premise of *p* < 0.05, up-regulated gene was defined as logFC > 0.5, on the contrary, down-regulated gene was defined as logFC < -0.5. and Gene ontology (GO) and Kyoto encyclopedia of genes and genomes (KEGG) pathway enrichment analysis were visualized with R packages “limma”, “org.Hs.eg.db”, “clusterProfiler”, and “enrichplot” [16].

### Correlations of ARPC5 expression with immunity-related indexes

Stromal, immune, and ESTIMATE scores between glioma with high vs. low ARPC5 expression in TCGA database were calculated using the ESTIMATE algorithm. The data were assessed and visualized using the R packages “estimate” and “limma”. Then correlation analysis between ARPC5 expression and infiltrating immune cells of glioma in TCGA database were performed through R packages “limma”. The correlations of ARPC5 expression with tumor mutation burden (TMB) and microsatellite instability (MSI) in glioma were analyzed by spearman’s rank correlation method. We downloaded clinicaldata from The Cancer Immunome Atlas (TCIA), and the correlation between ARPC5 with immunotherapy was conducted and visualized with R packages “limma”, “ggpubr”.

### Cell culture and transfection

Glioblastoma U118MG, U87, A172, T98G, LN229, LN18 and U251 cells were purchased from the Chinese Academy of Sciences Cell Bank. LN229 and U251 cells were used to study the effect of ARPC5 on biological behavior of glioma cells. The interference sequence of ARPC5 sh1 is 5’-CCGGGTTCAATCTCTGGACAAGAATCTCGAGATTCTTGTCCAGAGATTGAACTTTTT-3’ and 5’-AATTAAAAAGTTCAATCTCTGGACAAGAATCTCGAGATTCTTGTCCAGAGATTGAAC-3’. The interference sequence of ARPC5 sh2 is 5’-CCGGCATTGTCTTGAAGGTGCTCATCTCGAGATGAGCACCTTCAAGACAATGTTTTT-3’ and 5’-AATTAAAAACATTGTCTTGAAGGTGCTCATCTCGAGATGAGCACCTTCAAGACAATG-3’.

### RNA isolation and qPCR

Total RNA was extracted from transfected LN229 and U251 cells with Trizol reagent (Invitrogen). The total RNA quality of these samples was detected by Nanodrop and agarose gel electrophoresis. Used the transcriptor first-strand cDNA synthesis kit to synthesize complementary DNA. Used SYBR Green to perform quantitative PCR on Quant Studio 6 Flex. U6 is used for ARPC5 standardization. The relative multiples of ARPC5 expression were determined using relative quantitative methods. The primer sequence was ARPC5-F: 5’-AGTTCGTGGACGAAGAAGATG-3’, ARPC5-R: 5’-CCTGACTCTTGGTGTTGATAGG-3’, U6-F: 5’-CGCTTCGGCAGCACATATAC-3’, U6-R: 5’-AGGGGCCATGCTAATCTTCT-3’.

### MTT cell proliferation assay

MTT assay was performed to evaluate cell proliferation. LN229 and U251 cells were seeded in 96 well plates at the density of 3000 cells/100 µL/well. After inoculation for 24, 48 and 72 h, 10 µL of MTT (5 mg/ml) was added to each hole of the plate, and incubated at 37℃ for 4 h. Then, 150 µL DMSO was added to per hole, and incubated at 37℃ for 5 min, this process runs in a dark and oscillating manner. Finally, the absorbance was measure at 570 nm with a microplate reader.

### Clone formation assay

Clone formation assay was used to evaluate cell proliferation activity. LN229 and U251 cells were seeded in 6-well plates at the density of 1000 cells/ml/well. After LN229 cells were cultured for 10 days, discard the supernatant and fixed with 4% paraformaldehyde for 20 min. Then cells were washed with PBS for three times and added crystal violet stain solution for 20 min. Finally, dried the 6-well plate and obtained the image by the scanner.

### Transwell migration assay

Transwell assay was used to analyze the migration ability of LN229 and U251 cells. The cells were seeded in the transwell chamber at the density of 50,000 cells/well. The lower chamber was added culture medium containing 10% serum. After 24 h of culture, the cells were fixed with 4% paraformaldehyde and stained with 0.1% crystal violet. Cell migration was observed by optical microscope.

### Immunohistochemistry

Immunohistochemistry is basically as described previously. Glioma tissue samples and glioma chip ZL-BraG180sur01 were incubated with ARPC5 antibody (16717-1-AP, proteintech, Wuhan, China) and CD3 antibody (17617-1-AP, proteintech, Wuhan, China). Immunohistochemical evaluation was analyzed by QuPath software. Apply QuPath software to grade fluorescence intensity or DAB staining intensity to 4 levels to evaluate protein expression. The staining intensity score was as follows: 0, no staining; (1) Weak staining; (2) Moderate staining; (3) Strong staining.

### Western blotting

The total protein of glioma cells was extracted with RIPA buffer containing protease inhibitor. Measured the loading amount of each sample using BCA kit. Used 12% SDS-PAGE to separate 20 µg of protein. Subsequently, the protein was transferred to the PVDF membrane and blocked with 5% nonfat milk powder for 1 h. ARPC5 (1:600) and GAPDH (1:10000) were added and incubated overnight at 4℃. Finally, incubated the PVDF membrane with appropriate secondary antibody (1:2000) for 1 h. The expression level of ARPC5 protein was detected by chemiluminescent imaging system.

### Statistical analysis

All statistical analysis and visualization were conducted by language software (version 4.1.2). All RNA-sequencing data was normalized by log2 transformation. Survival analyses were conducted using the Kaplan-Meier curve and cox regression model in this study. The wilcoxon rank-sum test was used to assess correlations between the ARPC5 expression and corresponding clinical information. *p* < 0.05 was considered statistically significant.

## Results

### Abnormally high expression of ARPC5 in glioma

**Fig. 1:**
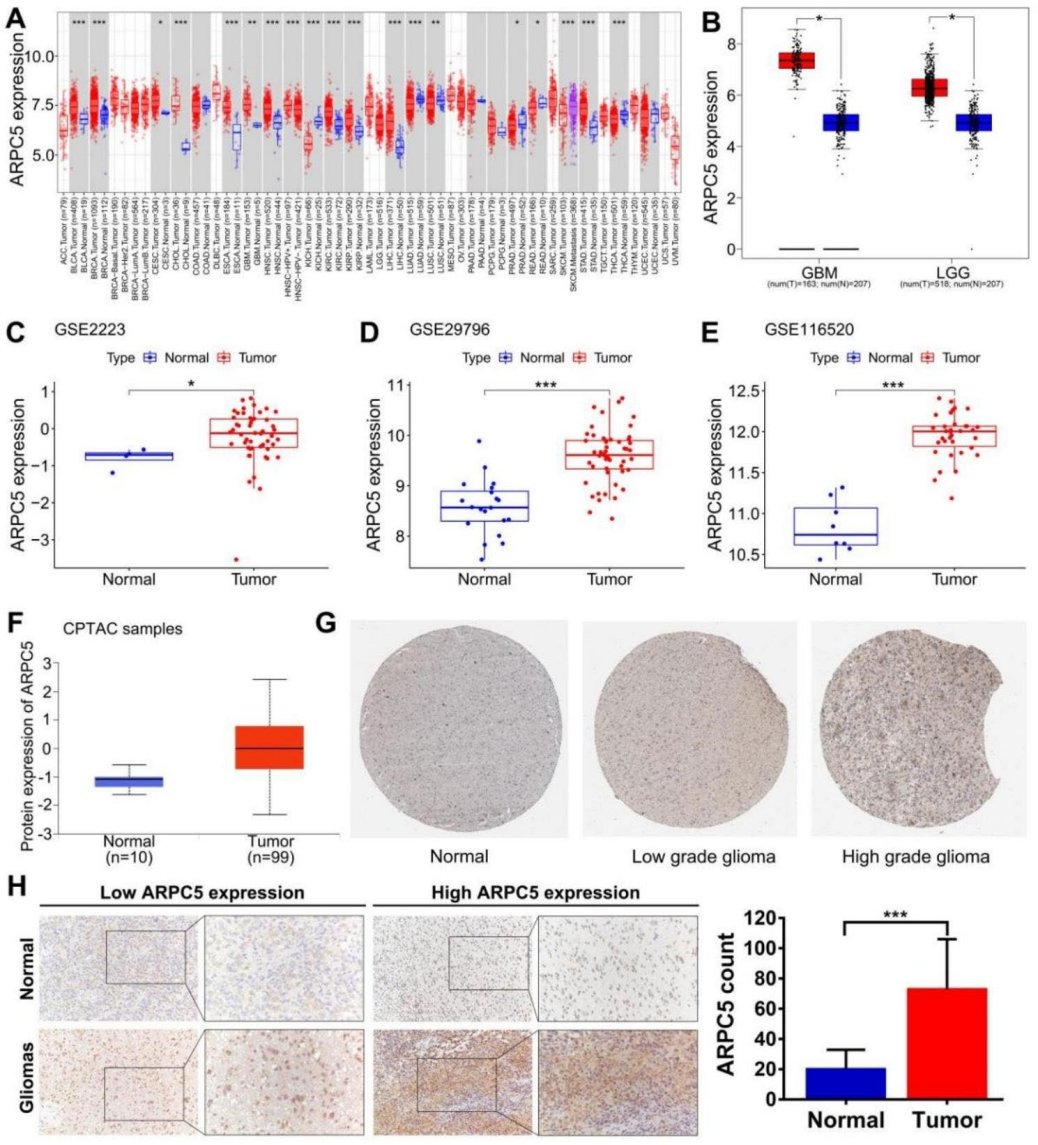
Abnormally high expression of ARPC5 in glioma. (**A**) Differential expression of ARPC5 in various tumor types was analyzed based on the TIMER online website. (**B**) The distinct upregulation of ARPC5 in glioma was demonstrated in GEPIA online website. (**C**) Box plot based on the expression of ARPC5 in the GSE2223 (Glioma = 50, Normal = 4). (**D**) Box plot based on the expression of ARPC5 in the GSE29796 (Glioma = 52, Normal = 20). (**E**) Box plot based on the expression of ARPC5 in the GSE116520 (Glioma = 34, Normal = 8). (**F**) Box plot based on the protein expression of ARPC5 in the CPTAC samples (Glioma = 99, Normal = 10). (**G**) ARPC5 protein expression was detected in cerebral cortex (Patient id: 1582), low grade glioma (Patient id: 3365), and high-grade glioma (Patient id: 3251) tissues from HPA dataset. (**H**) Representative images of ARPC5 expression in glioma and its surrounding tissues. *p < 0.05, **p < 0.01, ***p < 0.001

Some studies have reported the critical role of the Arp2/3 complex in glioma cell invasion and migration, it is still unclear which components of the Arp2/3 complex affect the clinical prognosis in the patients. We first examined the expression of the Arp2/3 complex in glioma. Subsequently, ARPC5 was screened according to the criterion that whether AUC value was greater than 0.7 in CGGA database (Supplementary Fig. 1). In order to further analyze the expression of ARPC5 in tumor tissues, we retrieved the TIMER database and observed that ARPC5 was abnormally expressed in 19 tumor types, including in GBM (Fig. 1A). To ensure the factuality of the above result, we used GEPIA, GSE2223, GSE29796, and GSE116520 datasets to verify that ARPC5 was highly expressed in gliomas, compared with normal brain specimens, as presented in Fig. 1B-E. Moreover, elevated ARPC5 protein level is detected in the CPTAC and HPA database (Fig. 1F-G). In addition, we evaluated glioma and near-by tissues of 10 patients by immunohistochemistry tests, and the results indicated that ARPC5 expression was obviously higher in glioma tissues (Fig. 1H). Similar results were also found in the Western blot analysis (Supplementary Fig. 2A). In general, the results above suggested that ARPC5 was abnormally expressed in gliomas.

### Relationship between ARPC5 gene expression and clinical characteristic

Subsequently, we further explored whether ARPC5 upregulation was associated with clinicopathological features of patients in CGGA database. The results showed that distinct expression of ARPC5 significantly correlated with gender, WHO classification, chemo status, IDH mutation status, 1p/19q codeletion status, MGMTp methylation status, PRS type, and histological type (Supplementary Fig. 3).

### Correlation between the different status of ARPC5 expression and prognosis of glioma patients

**Fig. 2:**
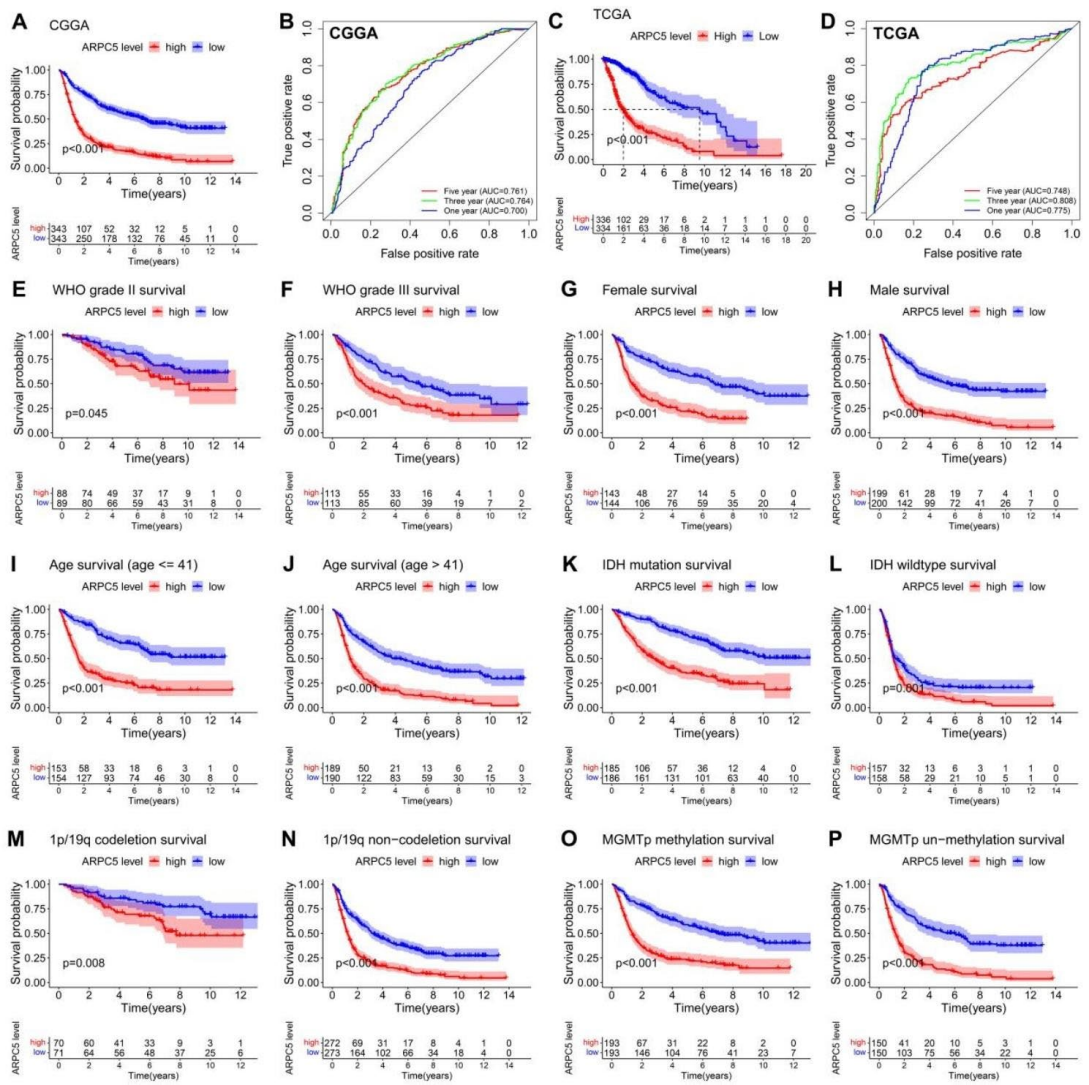
Correlation between different status of ARPC5 expression and prognosis of glioma patients. (**A**) Survival analysis of ARPC5 in CGGA database. (**B**) ROC curve analysis of ARPC5 at 1, 3, and 5 years in CGGA database. (**C**) Survival analysis of ARPC5 in TCGA database. (**D**) ROC curve analysis of ARPC5 at 1, 3, and 5 years in TCGA database. Survival analysis of the signature in patients stratified by grade (**E, F**), gender (**G, H**), age (**I, J**), IDH mutation status (**K, L**), 1p/19q codeletion status (**M, N**), and MGMTp methylation status (**O, P**) in CGGA database

**Fig. 3:**
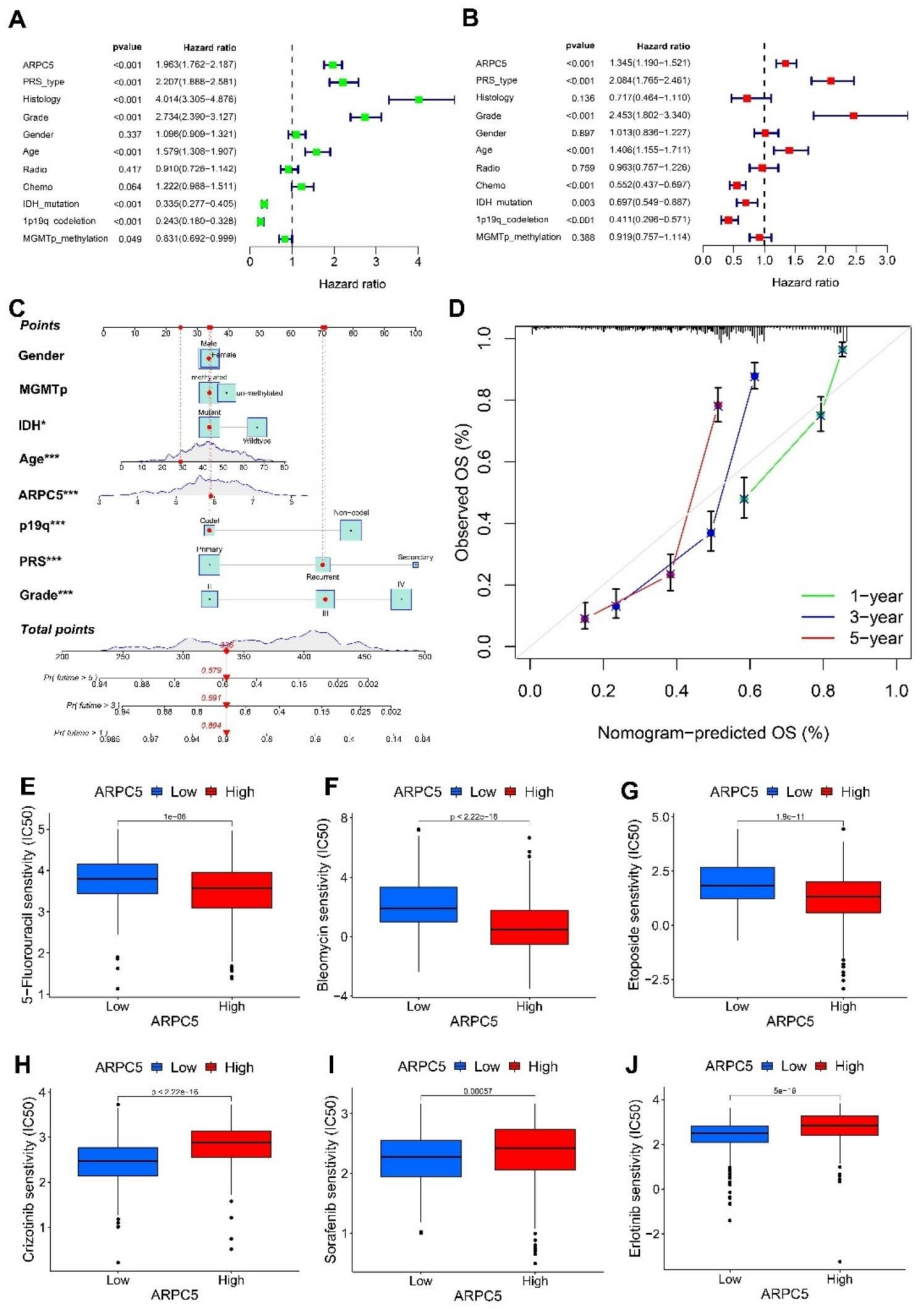
Prognostic significance of ARPC5 and its association with drug sensitivity in glioma. (**A**) Univariate regression analysis of prognosis in CGGA database. (**B**) Multivariate analysis of prognosis in CGGA database. (**C**) The nomogram to predict the association between ARPC5 expression and OS was developed using the CGGA dataset. (**D**) The calibration curve for the nomogram-predicted OS. ARPC5 affects the sensitivity to 5-fluorouracil (**E**), bleomycin (**F**), etoposide (**G**), crizotinib (**H**), sorafenib (**I**), and erlotinib (**J**)

Although it has been elucidated that ARPC5 expression was abnormally increased in glioma and closely related to different clinical characteristics, the prognostic values of ARPC5 in glioma patients were still unclear. Therefore, we investigated the impact of ARPC5 on the overall survival of glioma patients, using the CGGA and TCGA datasets. Kaplan-Meier survival analysis showed that high expression of ARPC5 was significantly correlated with poor prognosis in the CGGA database (Fig. 2A). The validity of the results has been verified through the TCGA database (Fig. 2C). In addition, the AUC values for ARPC5 to predict 1-, 3- and 5-year survival were 0.700, 0.764, and 0.761, respectively, in the CGGA database (Fig. 2B). The ROC curve of ARPC5 for 1-, 3-, and 5- year outcomes had AUC values of 0.775, 0.808, and 0.748, respectively, in the TCGA database (Fig. 2D). To examine the association between ARPC5 expression and glioma prognosis in detail, we divided the glioma patients into different groups through different molecular subtypes for grade (WHO 2, WHO 3), gender (female, male), age (age ≤ 41, age > 41), IDH (mutant, wild type), 1p/19q (codel, non-codel), and MGMT (methylated, un-methylated) status (Fig. 2E-P). The results showed that highly expressed ARPC5 was significantly correlated with poor prognosis in various subgroups. Moreover, regression analysis showed that ARPC5 expression, PRS type, grade, and age might be independent risk factors for poor prognosis of glioma patients. In comparison, IDH mutation and 1p/19q codeletion status may represent protective factors (Fig. 3A-B). In addition, we established a nomogram containing the prognostic factors, such as gender, MGMT status, IDH status, age, ARPC5 expression, 1p19q status, PRS, and grade, to forecast the possibility of 1-, 3-, and 5-year OS in glioma patients. For example, a patient with a total point of 336 would have 1-, 3-, and 5-year survival rates were 89.4%, 69.1%, and 57.9%, respectively (Fig. 3C). Furthermore, our data indicated that nomogram-predicted survival is closed to actual survival (Fig. 3D). ARPC5 was also found to be associated with the sensitivity of multiple drugs, including 5-fluorouracil, bleomycin, etoposide, crizotinib, sorafenib, and erlotinib (Fig. 3E-J). These data indicated that ARPC5 may serve as a potential prognostic factor for glioma.

### Enrichment analysis identifies ARPC5-associated signaling pathways

**Fig. 4:**
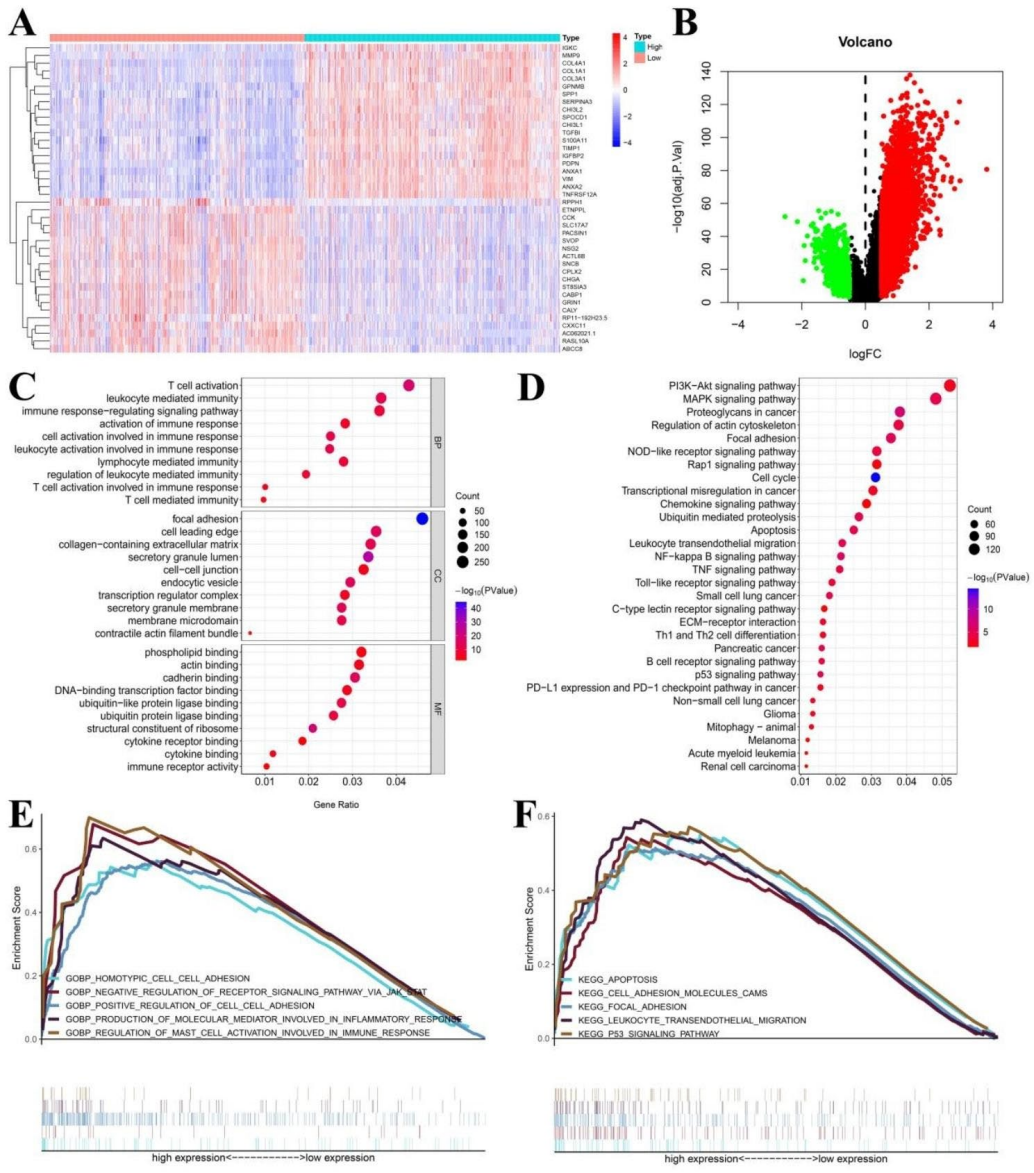
Enrichment analysis identifies ARPC5-associated signaling pathways. (**A**) Heatmap for DEGs generated by compare the difference of ARPC5 expression in glioma from the CGGA dataset. (**B**) Volcano plots illustrated all DEGs. Barplot showing the GO (**C**) and KEGG (**D**) analysis for ARPC5 in glioma. (**E**) GO functional annotation of ARPC5 in glioma from GSEA. (**F**) KEGG pathway analysis of ARPC5 in glioma from GSEA.

To further validate the effect of ARPC5 on glioma, we conducted a series of enrichment analyses. Based on CGGA database, the heatmap showed the 40 genes with the most significant differences in positive and negative correlation (Fig. 4A). Differentially expressed gene were displayed on a volcano map (Fig. 4B). GO enrichment analysis also showed that ARPC5 were enriched in immune regulatory pathways, including T cell activation, leukocyte mediated immunity, immune response-regulating signaling pathway, activation of immune response, cell activation involved in immune response, leukocyte activation involved in immune response, lymphocyte mediated immunity, regulation of leukocyte mediated immunity, T cell activation involved in immune response, and T cell mediated immunity (Fig. 4C). KEGG pathway analysis showed that ARPC5 were mainly enriched in multiple tumor-related signaling pathways, such as proteoglycans in cancer, small cell lung cancer, pancreatic cancer, non-small cell lung cancer, glioma, melanoma, acute myeloid leukemia, and renal cell carcinoma (Fig. 4D); the full information is included in Supplementary Table S1. GSEA was conducted to investigate signal pathways in the development of glioma and compare datasets with low and high ARPC5 expression. In GO functional annotation analysis, some immune and cell adhesion regulatory pathways were enriched, such as regulation of mast cell activation involved in immune response, production of molecular mediator involved in inflammatory response, production of molecular mediator involved in inflammatory response, and positive regulation of cell adhesion (Fig. 4E). In KEGG pathway analysis, ARPC5 expression was positively correlated with apoptosis, focal adhesion, cell adhesion molecules cams, leukocyte transendothelial migration, and p53 signaling pathway (Fig. 4F).

### Investigations the expression of ARPC5 in glioma through single-cell analysis

**Fig. 5:**
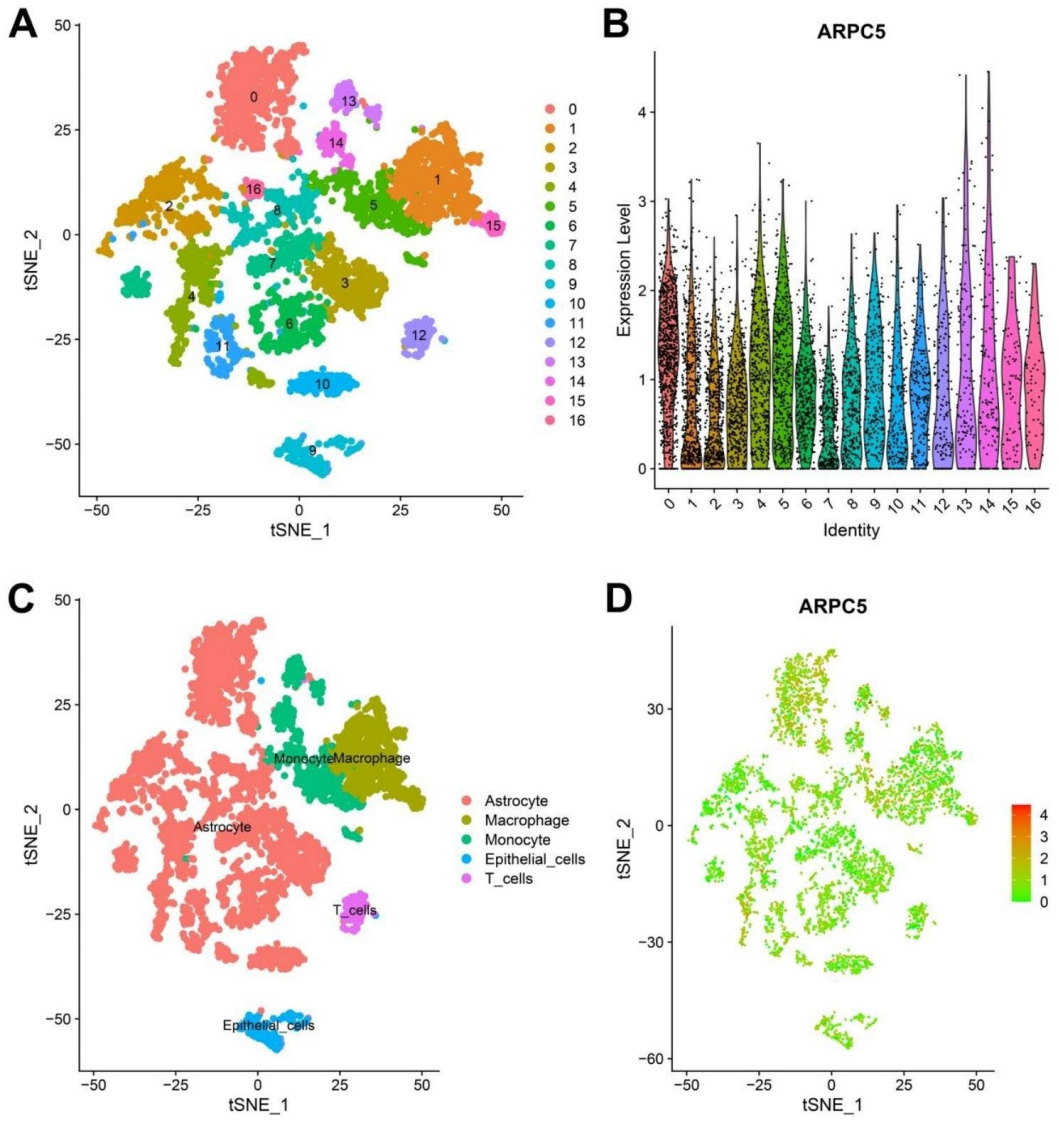
Investigations the expression of ARPC5 in glioma through single-cell analysis. (**A**) Through dimension reduction analysis of CGGA single-cell data, TSNE plot depicted that 6,148 cells were classified into 16 clusters. (**B**) The violin plot showed the expression of ARPC5 in each type of cluster. (**C**) Sixteen clusters were divided into 5 types of cells: astrocyte, macrophage, monocyte, epithelial and T cell. (**D**) The scatter plot shows the expression of ARPC5 in each cell

We further studied the distribution of ARPC5 expression in different types of gliomas by analyzing single-cell data. 6,148 cells from gliomas were classified into 16 clusters (Fig. 5A). In addition, we also examined the expression of ARPC5 in different clusters (Fig. 5B). Subsequently, according to the characteristics of different clusters, all cells were divided into five cell populations. The results showed that ARPC5 expression was significantly increased in astrocytes, monocytes and T cells, which indirectly indicated that ARPC5 might participate in immune cell infiltration (Fig. 5C-D).

### Correlations of ARPC5 expression with immunity-related indexes

**Fig. 6:**
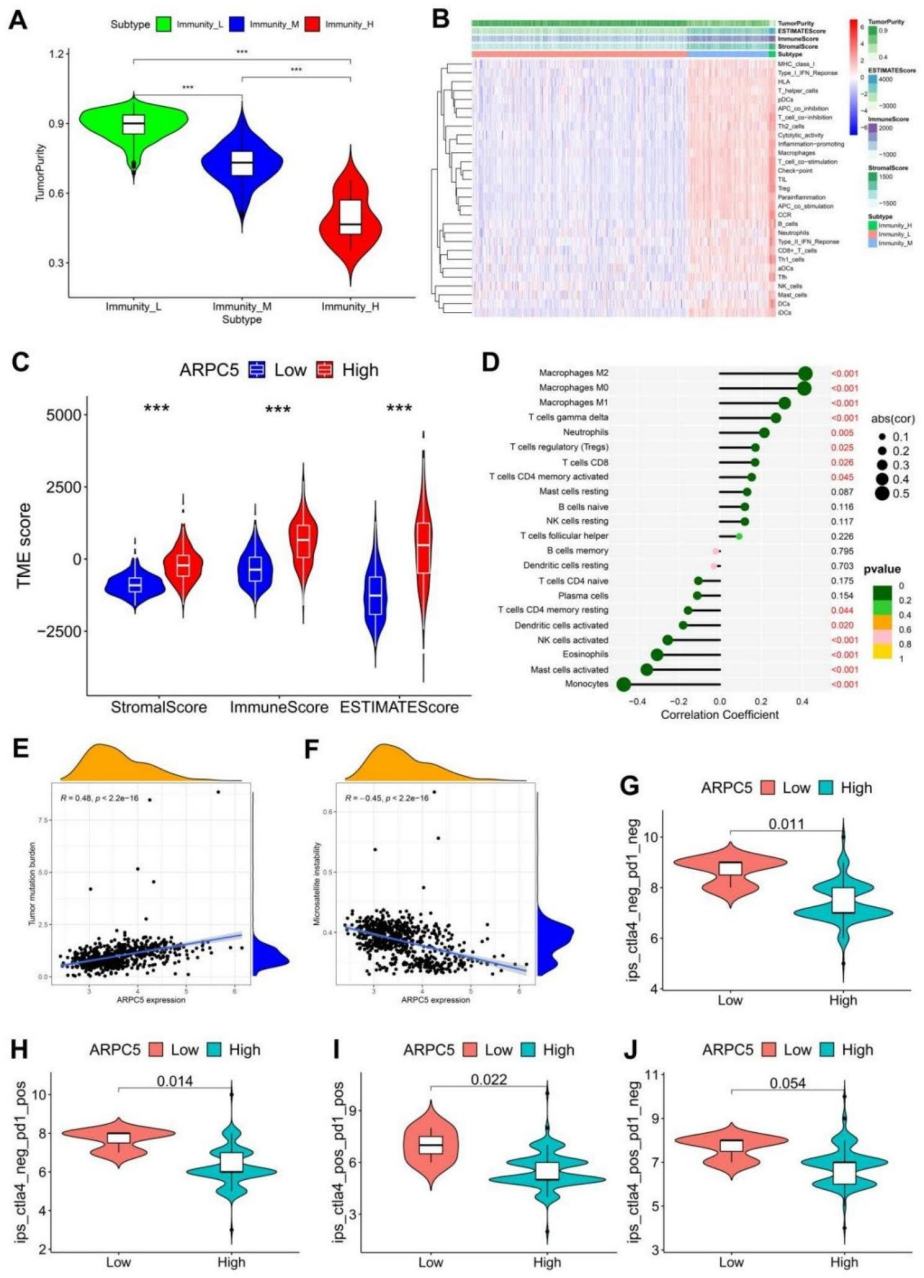
Correlations of ARPC5 expression with immunity-related indexes. (**A**) Violin plot showed the correlation between immune classification and tumor purity. The immune infiltration models of low- and high-ARPC5 were detected through ssGSEA methods in glioma from the TCGA database. (**C**) Violin plot showed the correlation between ARPC5 with stromal scores, immune scores, and ESTIMATE scores. (**D**) Lollipop showed the correlation between ARPC5 with infiltrating immune cells. Scatter plot showed the correlation between ARPC5 with TMB (**E**) and MSI (**F**). (G-J) Violin plot showed the correlation between ARPC5 with immunotherapy

The patients were divided into low immunity group, medium immunity group, and high immunity group according to their immune cell infiltration status (Fig. 6A). The results showed that ARPC5 expression is positively correlated with the infiltration of immune cells (Fig. 6B). We calculated the relationship between immune microenvironment and ARPC5 expression in glioma via the estimate algorithm, and the results found that high expression of ARPC5 was positively correlated with immune score (Fig. 6C). Furthermore, the expression of ARPC5 was positively correlated with macrophages, gamma delta T cells, neutrophils, regulatory T cells, CD8^+^ T cells, activated memory CD4^+^ T cells. However, it was negatively correlated with the infiltrating memory resting CD4^+^ T cells, activated dendritic cells, activated NK cells, eosinophils, activated mast cells, and monocytes (Fig. 6D). In addition, TMB and MSI were performed to predict the immunotherapy role of ARPC5 in glioma. Results showed that ARPC5 expression was obviously positively related to TMB (Fig. 6E); while negatively related to MSI (Fig. 6F). Our results also showed that ARPC5 expression is positively correlated with most immune markers in glioma (Supplementary Fig. 2C). Meanwhile, further studies showed that high expression of ARPC5 was related to the poor immunotherapy efficacy of glioma (Fig. 6G-J).

### Effect of ARPC5 on the expression of CD3 and prognosis

**Fig. 7:**
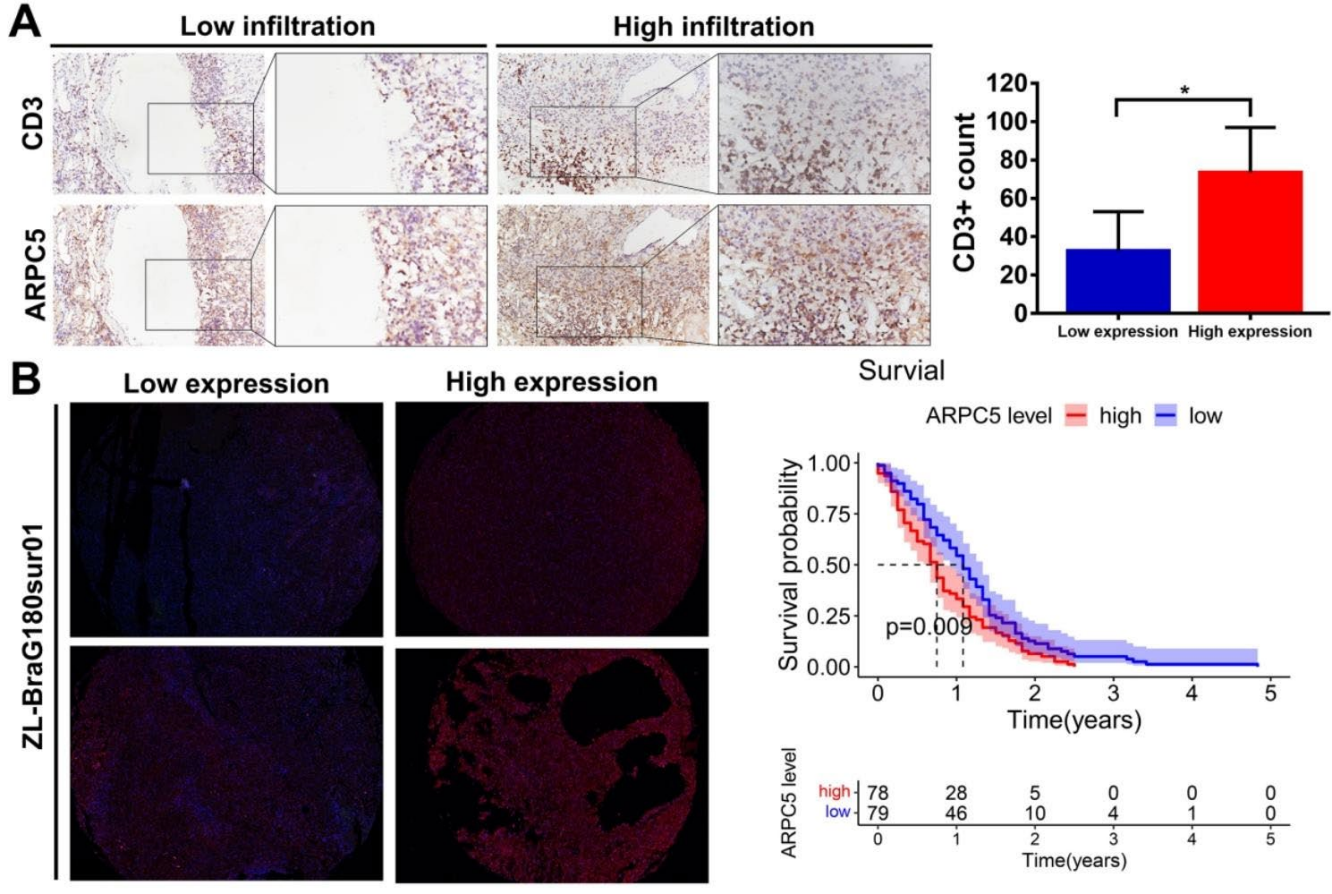
Effect of ARPC5 on the expression of CD3 and prognosis. (**A**) Representative images revealed the relationships between the ARPC5 expression and T cell marker CD3 in gliomas. (**B**) Survival analysis of ARPC5 in glioma chip ZL-BraG180sur01.

In order to further verify the results of the above bioinformatics analysis, the relationship between ARPC5 and T cell marker (CD3) was evaluated by immunohistochemistry. Our results revealed that there was a significant positive correlation between ARPC5 expression and T cells in 10 glioma samples (Fig. 7A). Then, we further used the glioma chip and found that the expression of ARPC5 is closely related to the prognosis of glioma patients (Fig. 7B).

### Knockout of ARPC5 can significantly inhibit the proliferation and migration of LN229 and U251 cells

**Fig. 8:**
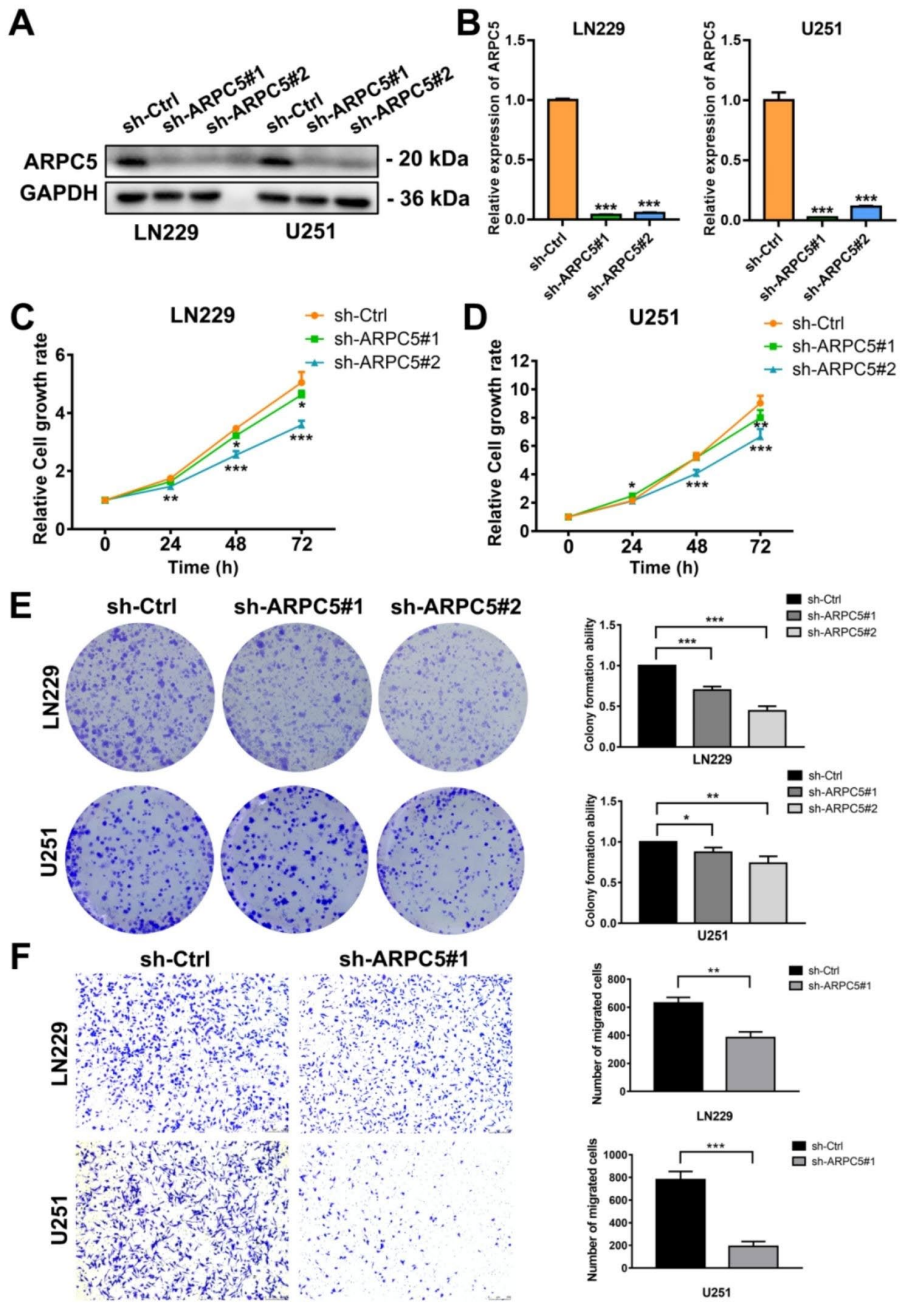
Effect of ARPC5 on the proliferation and migration of glioma cells. (**A**) The expression level of ARPC5 protein was detected in LN229 and U251 cells after shRNA interference, GAPDH was used to confirm equal protein loading. (**B**) RT-qPCR verified the expression efficiency of ARPC5 in LN229 and U251 cells after shRNA interference. Growth curve assessing the effect of ARPC5 on the proliferation of LN229 (**C**) and U251 (**D**) cells. (**E**) The representative image showed the clone formation ability of LN229 and U251 cells after transfection. (**F**) Transwell analysis was further used to examine the effect of ARPC5 on migration of LN229 and U251 cells, and the number of migrating cells was quantitatively analyzed. *p < 0.05, **p < 0.01, ***p < 0.001

To investigate the function of ARPC5 in glioma cell lines, a variety of phenotypic experiments were performed. First, we detected the protein expression level of ARPC5 in glioma cell lines by Western blot, and found that ARPC5 expression in LN229 and U251 cells was significantly higher than that in other glioma cell lines, which was suitable for subsequent experiments (Supplementary Fig. 2B). Subsequently, the inhibitory effects of ARPC5-specific shRNA on the expression of ARPC5 in LN229 and U251 cells were detected by Western blot and RT-qPCR (Fig. 8A-B). MTT and clone formation methods were used to determine the effect of ARPC5 on the proliferation of LN229 and U251 cells, and the results showed that ARPC5 knockout could obviously inhibit cell proliferation (Fig. 8C-E). In addition, the role of APRC5 in glioma cells migration was evaluated by transwell assays. Compared with the control group, the migration ability of LN229 and U251 cells transfected with shARPC5 was significantly reduced (Fig. 8F).

## Discussion

Malignancies of the brain with an estimated 308,102 new cases account for 1.6% of all cancer cases, and its morbidity has increased over recent years [17, 18]. Gliomas represent 80% of brain malignancies; despite recent advances in diagnostic and therapeutic measures, the prognosis of glioma patients remains poor [19]. For example, the predicted median survival rate of glioblastoma multiforme (grade IV) is 12–15 months, and those of anaplastic glioma (grade III) is 2–5 years [20]. In recent years, the recommendation of molecular diagnostics has received attention in gliomas, which could play an essential role in clinical practice for the evaluation of prognosis and the selection of suitable treatment [21]. Moreover, with the development of high-throughput sequencing technology and resulting knowledge about genetic alterations occurring in gliomas, new biomarkers are expected to improve the prognosis of glioma patients.

In this research, we first analyzed the expression of ARPC5 in glioma on account of the TCGA database, which verified that ARPC5 was upregulated in glioma patients. The GEO and UALCAN online analyses further demonstrated these results. Previous studies have demonstrated that the upregulation of ARPC5 can be found in a variety of tumors, such as head and neck squamous cell carcinoma and multiple myeloma, in agreement with our observational results [22, 23]. Second, we studied the clinical significance of ARPC5 expression changes in glioma patients and found that high expression level of ARPC5 were significantly related to several clinical features, such as gender, histological grade, chemotherapy status, relapses, IDH mutation, 1p/19q codeletion, and MGMTp methylation, suggesting that its overexpression may play a catalytic role in the clinical progress of glioma. The Kaplan-Meier curves revealed that high ARPC5 expression predicts a poor prognosis for patients with grade II and III gliomas, and the same result was obtained for different molecular subtypes, such as gender, age, IDH mutation status, 1p/19q co-deletion status, and MGMT methylated status. However, it should be pointed out that no significant differences between ARPC5 expression and poor prognosis in grade IV gliomas. One possible explanation about these results is that intratumoral molecular heterogeneity in glioblastoma undermines robust and durable responses to treatment [24, 25]. ROC curve, univariate and multivariate analyses, and a clinical correlation nomogram were performed to further indicate that ARPC5 was an important predictor of glioma. Thus, based on the above studies, ARPC5 may serve as an oncogene in gliomas from different viewpoints and represent a risk factor for poor prognosis, but the pathological mechanism is yet to be elucidated.

In order to explore the mechanism by which ARPC5 may influence prognosis in patients with glioma, we performed enrichment analysis to conclude the ARPC5-related genes were involved in cancer-related cell signaling pathways, such as immune, cell adhesion, and apoptosis. Immunotherapy manipulates the immune system to target tumor cells with fewest adverse effects and suppress tumor development [26]. Glioma is considered to be an “immune-privileged” tumor with complex tumor microenvironment [27]. Despite immunotherapy has made significant progress in various cancers, on account of the selectivity of the blood-brain barrier and the lack of lymphatic involvement, the application of immunotherapeutic drugs is greatly limited in glioma [28]. Therefore, it is of great significance to study new molecular biomarkers and special immune status of glioma for tumor treatment. To further explore the relationship between ARPC5 and immune, we analyzed the effect of ARPC5 expression on tumor microenvironment. Our data first showed that high expression of ARPC5 was positive associated with immune score, stromal score and TMB in glioma. In addition, higher levels of ARPC5 expression were significantly correlated with macrophages, T cells, neutrophils, dendritic cells, NK cells, eosinophils, mast cells, and monocytes. Interestingly, In the tumor, macrophages can polarize into two different phenotypes, M1 phenotype activates T cells and subsequent adaptive immunity against brain tumor cells, while M2 polarization inhibits the production of immune factors and promotes tumor proliferation [29, 30]. Despite M1 and M2 macrophage are detected in the brain tumor, the immune function of M1 macrophage is impaired [31]. In addition, some studies have shown that activated M2 macrophage can transport breast cancer cells into the brain [32]. These results showed that the relationship between ARPC5 and immune cell infiltration is complex, and the further research needs to be focus on the synergy of multiple receptors involved in the immune response. Among immunotherapeutic approaches, immune checkpoint inhibitors (ICIs) have played a huge role in many types of cancers. TMB is a potential biological marker for prediction of the survival rate of patients treated with ICIs [33]. TMB levels may lead to modifications of proteins encoded by mutated genes [34]. Higher TMB level has advantage for responsive to ICIs therapy, which may increase the likelihood of these tumor cells being killed. However, inhibitory receptor interactions on immune cells are often preempted by tumors, thus leading to immune evasion. For example, the binding of PD-L1 to immune checkpoint PD-1, as well as the binding of CTLA-4 to CD80/CD86 ligands, are key inhibitory checkpoint signals that limit T cell activation and block its action on tumor cells [35, 36]. At present, CTLA-4 antibody or PD1 antibody have been approved by the U.S. Food and Drug Administration for clinical use [37]. Our data showed that higher ARPC5 expression was associated with higher TMB and lower immune response score in glioma, which meant that means that targeted inhibition of ARPC5 may become an attractive immunotherapy strategy in the treatment of glioma.

Due to the existence of molecular heterogeneity, different and sufficient targeted therapy may open up more possibilities for the treatment of glioma. Currently, the combination of immunotherapy, tumor microenvironment and several effective targeted therapies has attracted more and more attention [38]. Thus, we evaluated whether ARPC5 expression was correlated to drug sensitivity. As a result, we noted that the estimated IC50 of 5-fluorouracil, bleomycin, and etoposide in the high-risk group was significantly lower than that in the low-risk group, indicating that high-risk subpopulations presented higher sensitivity to above drugs. Meanwhile, the low-risk subgroup was more sensitive to crizotinib, sorafenib, and erlotinib. Each of the above drugs showed different degrees of anti-tumor properties. For example, 5-fluorouracil, an analog of the pyrimidine uracil, is an antimetabolite anticancer agent used for brain cancer treatment. Previous studies have confirmed that compound agents of 5-fluorouracil may cause tumor cell death and change immune cell infiltrate, leading to T cell mediated antitumor immune response [39, 40]. In addition, erlotinib is a tyrosine kinase inhibitor and may be used as a second line therapeutic agent for glioma treatment [41]. A combination of erlotinib, bevacizumab, and irinotecan is safe and well tolerated, and the median OS of patients is improved, especially for high-risk patients [42]. This indirectly proves the significance of ARPC5 in glioma treatment and indicates its prospect for final clinical application.

Collectively, we found that ARPC5 is overexpressed in gliomas, and its expression is closely related to various clinical characteristics of glioma patients. Meanwhile, a high ARPC5 expression could predict a poor prognosis in patients with gliomas. The present findings also indicated that ARPC5 may regulate glioma development by participating in tumor immunity. The researches preliminarily suggest that ARPC5 could act as a potential prognostic target in glioma.

### Electronic supplementary material

Below is the link to the electronic supplementary material.

Supplementary Material 1

Supplementary Material 2

Supplementary Material 3

Supplementary Material 4

Supplementary Material 5

Supplementary Material 6

## Data Availability

The datasets presented in this study can be found in the following online publicly available data sets: TCGA (https://cancergenome.nih.gov), CGGA (http://www.cgga.org.cn/), GEO (https://www.ncbi.nlm.nih.gov/geo/), HPA (https://www.proteinatlas.org/), UALCAN (https://ualcan.path.uab.edu/), TCIA (https://www.cancerimagingarchive.net/). All data Accessed on 27 March 2023.
