# Supplementary figures and images for: ARPC5 acts as a potential prognostic biomarker that is associated with cell proliferation, migration and immune infiltrate in gliomas
Source: BMC Cancer. 2023 Oct 3;23:937. doi: 10.1186/s12885-023-11433-w (PMC10548738; doi:10.1186/s12885-023-11433-w)

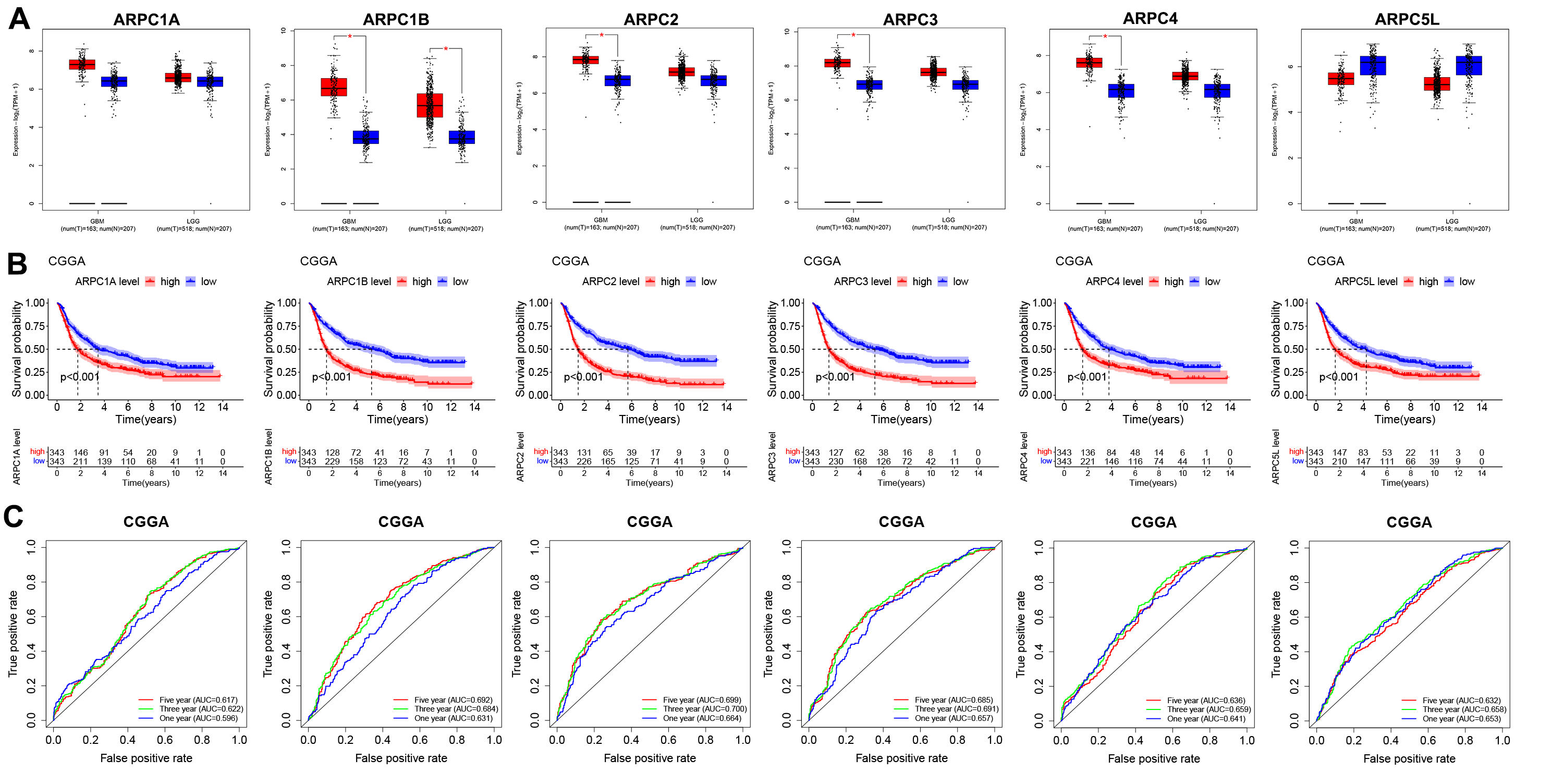

Supplement: Supplementary file 1 — Supplementary Material 1 [file 12885_2023_11433_MOESM1_ESM.tif]

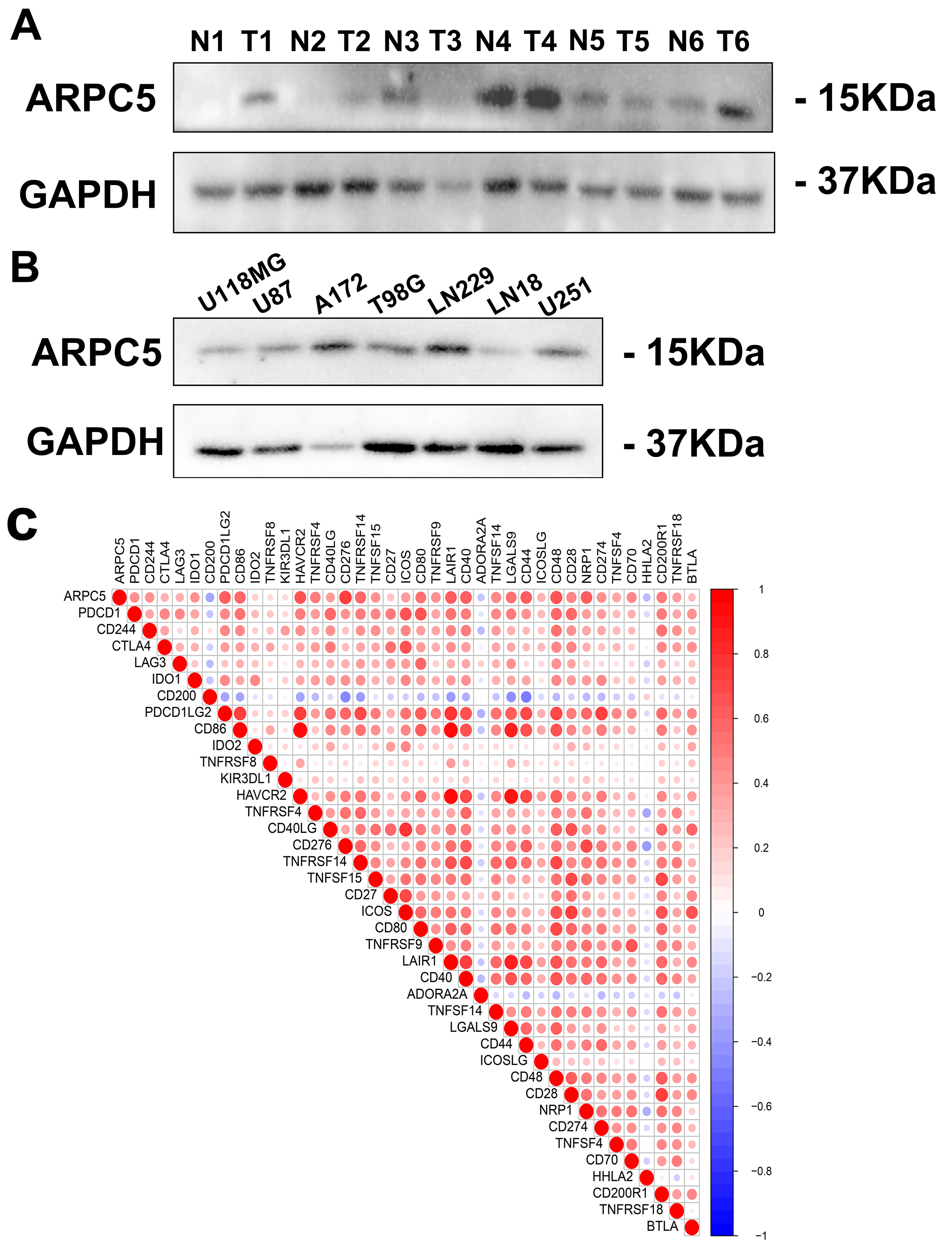

Supplement: Supplementary file 2 — Supplementary Material 2 [file 12885_2023_11433_MOESM2_ESM.tif]

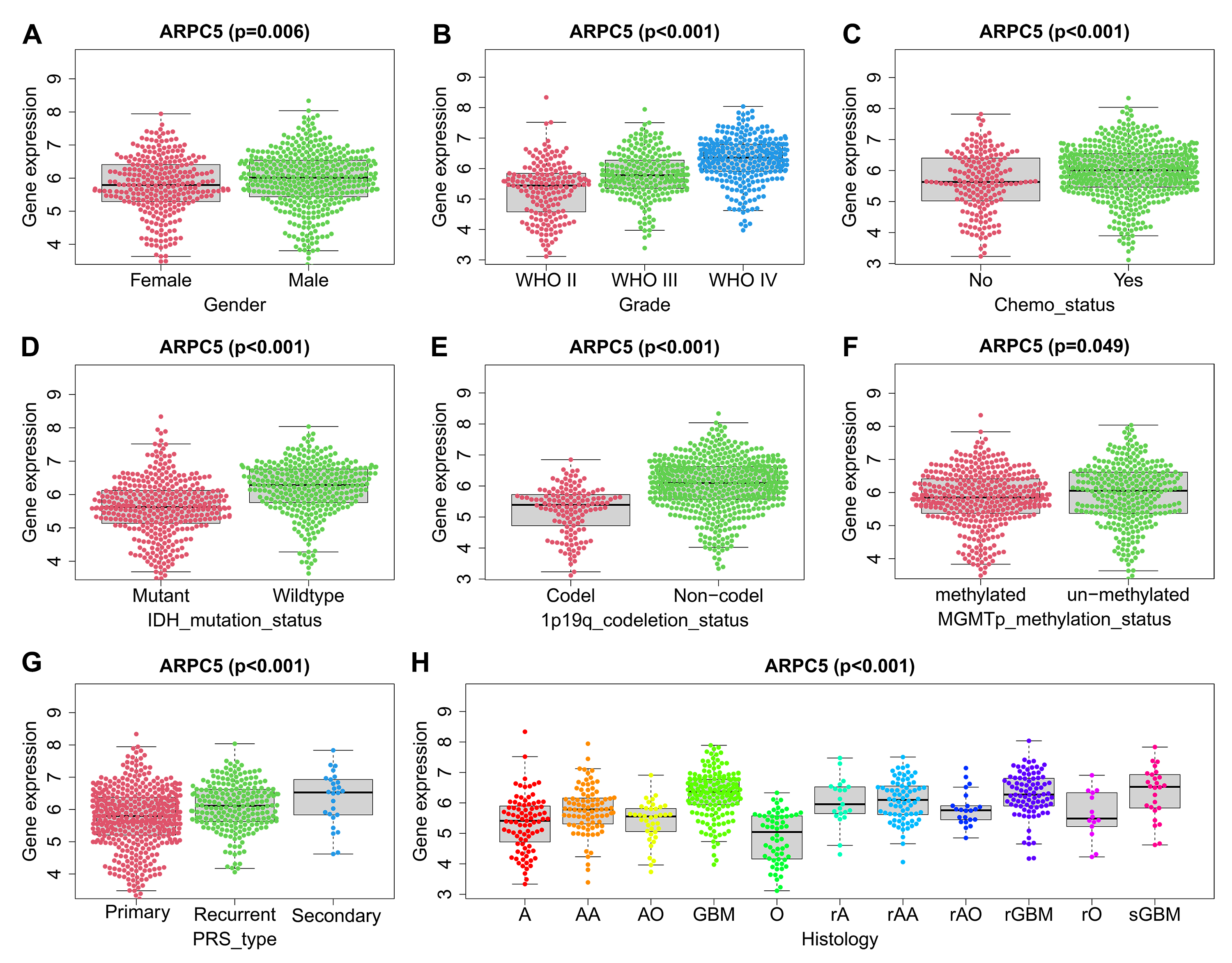

Supplement: Supplementary file 3 — Supplementary Material 3 [file 12885_2023_11433_MOESM3_ESM.tif]

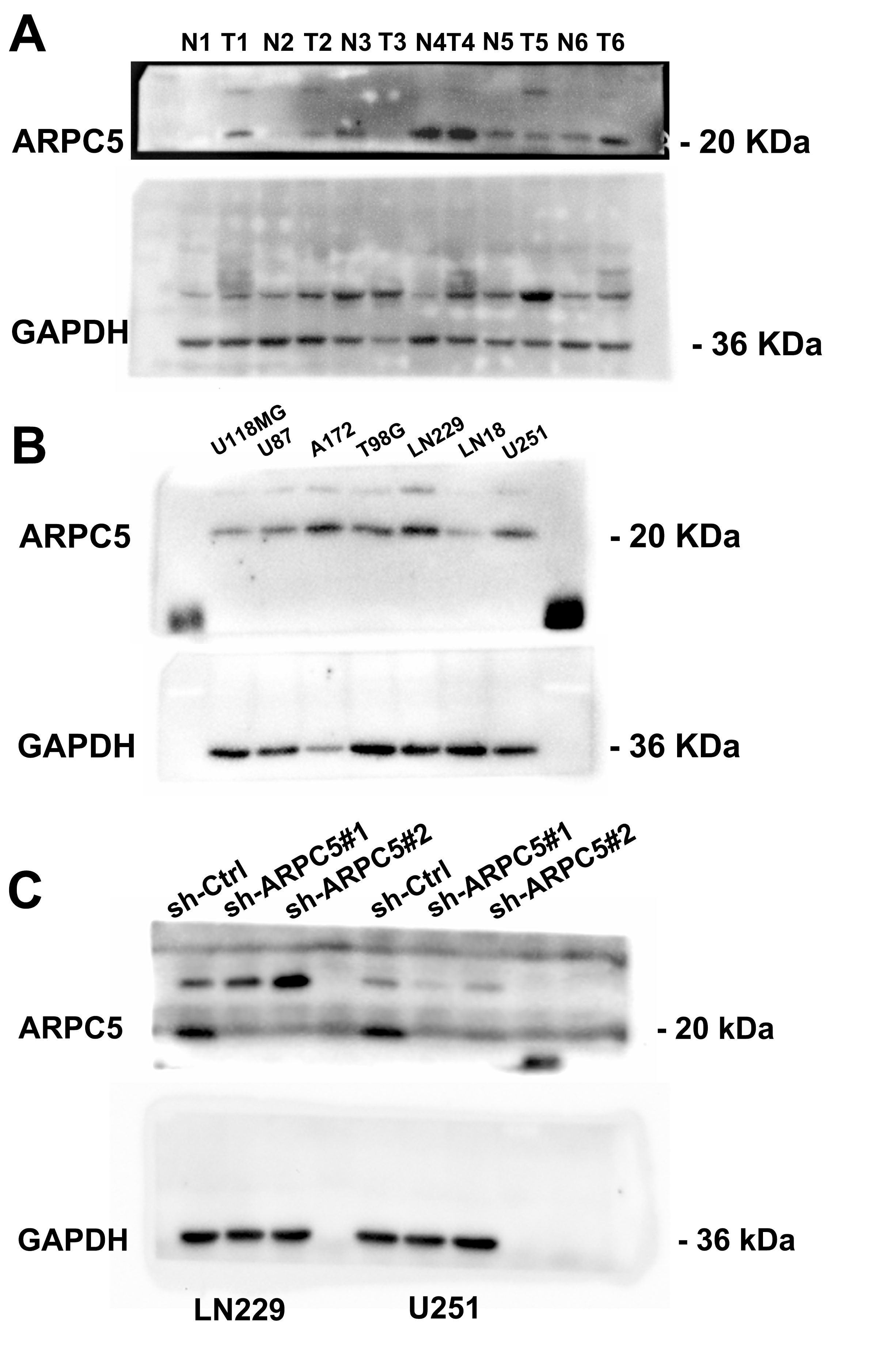

Supplement: Supplementary file 4 — Supplementary Material 4 [file 12885_2023_11433_MOESM4_ESM.tif]
